# Variation in mobility and exercise adaptations between *Drosophila* species

**DOI:** 10.1007/s00359-020-01421-x

**Published:** 2020-04-25

**Authors:** Tyler Cobb, Alyson Sujkowski, Courtney Morton, Divya Ramesh, Robert Wessells

**Affiliations:** 1 Department of Physiology, Wayne State University School of Medicine, Detroit, MI 48201 USA; 2 Department of Kinesiology, Wayne State University, Detroit, MI 48201 USA; 3 Department of Biology, University of Konstanz, 78464 Konstanz, Baden Württemberg Germany

**Keywords:** Drosophila, Mobility, Exercise, Octopamine

## Abstract

Locomotion and mobility have been studied extensively in *Drosophila melanogaster* but less is known about the locomotor capacity of other *Drosophila* species, while the response to chronic exercise in other species has yet to be examined. We have shown that adult male *D. melanogaster* adapt to exercise training with improved running endurance, climbing speed, and flight ability compared to unexercised flies. Here, we examine baseline mobility of *D. sechellia*, *D. simulans*, and *D. virilis*, and their response to chronic exercise training. We found significant interspecific differences in mobility and in the response to exercise. Although there is a significant sex difference in exercise adaptations in *D. melanogaster*, intraspecific analysis reveals few sex differences in other *Drosophila* species. As octopamine has been shown to be important for exercise adaptations in *D. melanogaster,* we also asked if any observed differences could be attributed to baseline octopamine levels. We find that octopamine and tyramine levels have the same rank order as baseline climbing speed and endurance in males, but do not predict the response to chronic exercise in males or females. Future research should focus on determining the mechanisms responsible for the inter- and intraspecific differences in mobility and the response to exercise.

**Electronic supplementary material:**

The online version of this article (10.1007/s00359-020-01421-x) contains supplementary material, which is available to authorized users.

## Introduction

With worldwide distribution, *Drosophila* are subject to different environmental challenges, which can significantly influence locomotion and mobility (Dillon and Frazier 2006; Valente et al. 2007). Adaptation to the environment is crucial for reproduction and ultimately survival and is partly dependent on locomotor capacity (Wcislo 1989). Since many *Drosophila* species are distributed in diverse areas (Throckmorton 1975), it is likely that their locomotor abilities have adapted to meet the demands of their environments. Indeed, many groups have found intra- and interspecific differences in locomotion and activity. Locomotor activity studies have often been conducted in the context of circadian rhythm (Thackeray 1989; Rogers et al. 2004; Vanlalhriatpuia et al. 2007; Bahn et al. 2009; Kauranen et al. 2012), while others have focused on mobility in the absence of circadian rhythm or under thermal stress (Bettencourt et al. 2009; Kjærsgaard et al. 2010; Berman et al. 2014). These studies highlight significant intra- and inter-specific differences but the differences in other aspects of mobility, such as climbing speed, running endurance, and flight ability, remain unknown.

*Drosophila melanogaster* is emerging as a suitable model organism to study the effects of endurance exercise which is accomplished by taking advantage of their negative geotaxis behavior (Piazza et al. 2009; Watanabe and Riddle 2017; Lowman et al. 2018). These models have shown that exercise improves parameters of metabolic health, mobility, cardiac performance, and upregulates metabolic and mitochondrial proteins (Piazza et al. 2009; Tinkerhess et al. 2012; Mendez et al. 2016). They have also been used to study the interaction of exercise and human diseases (Bajracharya and Ballard 2018; Damschroder et al. 2018b). We have recently established that the response to exercise requires the activation of octopaminergic (OA-ergic) neurons and is sexually dimorphic (Sujkowski et al. 2017). Exercised male flies have longer endurance, faster climbing speed, and better flight ability compared to unexercised siblings. In a separate exercise model, exercise increased activity levels and altered lipid metabolism, which was dependent on genotype and sex (Watanabe and Riddle 2017). Since there are clear intra- and interspecific differences in both locomotion and activity, we examined the differences in mobility and response to exercise training in different *Drosophila* species.

Here, we sought to explore the differences in baseline endurance, climbing speed, and flight ability between *D. sechellia*, *D. simulans*, and *D. virilis*. We also examined the response to an exercise training protocol using these mobility assays as outputs. Lastly, since the activation of OA-ergic neurons is required for exercise adaptations and is implicated in locomotion in *D. melanogaster*, we questioned if any of the observed differences could be attributed to baseline octopamine (OA) levels.

## Methods

### Fly stock and maintenance

Flies were maintained at 25 °C with a 12-h light–dark cycle and fed a 10% yeast 10% sucrose diet. *D. sechellia*, *D. simulans*, and *D. virilis* lines were generous gifts from Patricia Wittkopp.

### Exercise training and mobility assays

Exercise training and mobility assays were carried out as previously described (Damschroder et al. 2018a) with modifications.

#### Exercise training

Five-day-old flies were anesthetized using CO_2_ and collected into vials of 20 flies/vial which were separated into exercised (Ex) and unexercised (Un) groups. Vials of flies were placed on a machine (Power Tower) (Piazza et al. 2009), which repeatedly induces negative geotaxis to simulate exercise daily. The ramped exercise protocol consisted of 5 days of consecutive exercise followed by 2 days of rest for 3 weeks. Flies were exercised 2 h per day for the first week, 2 1/2 h per day the second week, and 3 h per day on the third week. The Power Tower drops approximately every 7.5 s, or eight times per minute. The total number of drops throughout the protocol is equal to 18,000. Exercise and assessments were conducted at the same time each day. Unexercised flies were also placed on the machine but their plugs were pushed down into the vial to restrict movement. All flies were transferred into new food vials daily.

#### Endurance

Climbing endurance was assessed both pre- and post-exercise training to longitudinally measure both the baseline endurance and the changes during training. A minimum of six vials of exercised and unexercised flies were placed on the Power Tower, and stimulated to run until fatigue which was scored visually by the experimenter. A vial was called fatigued when 80% or more of the flies could no longer climb off the food (i.e., the abdomen must fully be off the food) after three consecutive drops. When a vial was fatigued, it was removed from the Power Tower and the time was recorded. The Power Tower was not stopped during scoring and remained on for the duration of the assessment. Initially, vials were checked every 30 min and more frequently during the log-phase. Data were analyzed in Prism (Graphpad Software, San Diego, CA) using a log-rank test for significance. For analytic purposes, each vial of 20 was scored as a single unit.

#### Climbing speed

Speed was assessed immediately before the start of the exercise training program, and again five times weekly over the course of the three week exercise protocol. Five vials of 20 flies were transferred into empty vials and placed in a single row of a vial rack against a white background. To stimulate negative geotaxis, the rack was tapped gently onto a counter to knock the flies to the bottom of vials. Two seconds after the flies dropped to the bottom a picture was taken. This was repeated to obtain four pictures per assessment day. Pictures were analyzed using ImageJ. Vial height was measured (from the bottom of the vial to the start of the plug) in each photo and the percentage of vial height climbed reached by the midpoint of each fly body was measured. Because there is typically an age effect on climbing speed across three weeks, exercised cohorts are compared to age-matched unexercised cohorts. The average climbing height for each vial was calculated. That value was then averaged across four repeated pictures to obtain a final average value for each vial. For analytic purposes, each vial was treated as an individual, e.g., four vials of 20 flies each are treated as an *n* of four biological replicates. To assess the longitudinal change in climbing speed during the 3 weeks of exercise, climbing speed was normalized to the first day of exercise. Data were graphed and analyzed in Prism (Graphpad Software, San Diego, CA). Baseline climbing speed was analyzed using Student’s *t* test or one-way ANOVA. Longitudinal change in climbing speed was analyzed using two-way ANOVA with a Bonferroni correction for multiple comparisons.

#### Flight

Acute flight performance was performed as previously described with modifications (Babcock and Ganetzky 2014). A large cast acrylic tube (90 cm in length) was attached to a ring stand and setup upright on the floor. A polycarbonate sheet was coated with a thin layer of Tangle-Trap and inserted into the acrylic tube. A second ring stand was placed on a counter above the first ring stand. A funnel was attached to the second ring stand with a polycarbonate cylinder (drop tube) affixed to the ring stand, which was inserted into the funnel. Both ring stands were aligned such that the apex of the funnel was aligned with the center of the large cast acrylic tube. Eight vials of 20 flies from each group were dropped into the drop tube which ejected flies into the cast acrylic cylinder. Flies reflexively fly toward the sides of the cast acrylic cylinder and stick to the Tangle-Trap. Flies that land higher in the cylinder are considered to have better acute flight ability than those that land lower in the tube (Babcock and Ganetzky 2014). The polycarbonate sheet was removed from the cylinder and affixed against a white background. A meter stick was placed below the sheet and used for scaling during analysis. The sheet was photographed and then analyzed in ImageJ to calculate the landing height of individual flies. These data were analyzed and graphed in Prism and a one-way ANOVA was used to test for significance.

#### Lysosomal activity

Fly dissections were performed as previously described (Vogler and Ocorr 2009). Exposed hearts and fat bodies were stained with 0.01 µM LysoTracker green for 45 s and washed three times with PBS. Hearts and fat bodies were removed from the flies and mounted onto a microscope slide using VectaShield. Ten samples were mounted per slide. Samples were then imaged using fluorescence microscopy. Images were analyzed in ImageJ to count the number of puncta per designated area of heart or fat tissue. Data were graphed in Prism and one-way ANOVA was used to test for significance.

### Mass spectrometry

Quantification of OA and TA content was done as previously described (Sujkowski et al. 2017). Briefly, for each species, five male or female heads, respectively, were pooled to make one sample. Triplicates of samples were processed by homogenizing the heads in 190 µL of acidified acetone, ascorbic acid and the respective internal standards. The stable isotope labeled internal standards were octopamine-*d*3 and tyramine-*d*4 (Medical Isotopes Inc., Pelham, USA). The supernatants were dried, reconstituted in borate buffer and derivatized using 6-aminoquinolyl-*N*-hydroxysuccinimidyl carbamate (AQC). Sample clean-up was done using solid-phase extraction (SPE) cartridges (Phenomenex, Inc. Hyderabad, India), and the elute was dried completely before reonstituting it in 2% acetonitrile containing 0.5% formic acid. The liquid chromatography and the electrospray ionization conditions were the same as in Sujowski et al. (2017) with the addition of the MRMs for the internal standards (parent ion *m/z* for OA-*d*3 is 327.17, for TA-*d*4 is 312.36). The calibration curves were made over a 64-fold concentration range with the highest concentration being 0.8 µg/mL. Quantitation was done using the Xcalibur software (version 2.2 SP1.48). Statistical analyses and plotting was done using R (3.4.0). Effect sizes, confidence intervals, and raw data are given in the supplemetary material.

## Results

### Mobility assessments

**Fig. 1:**
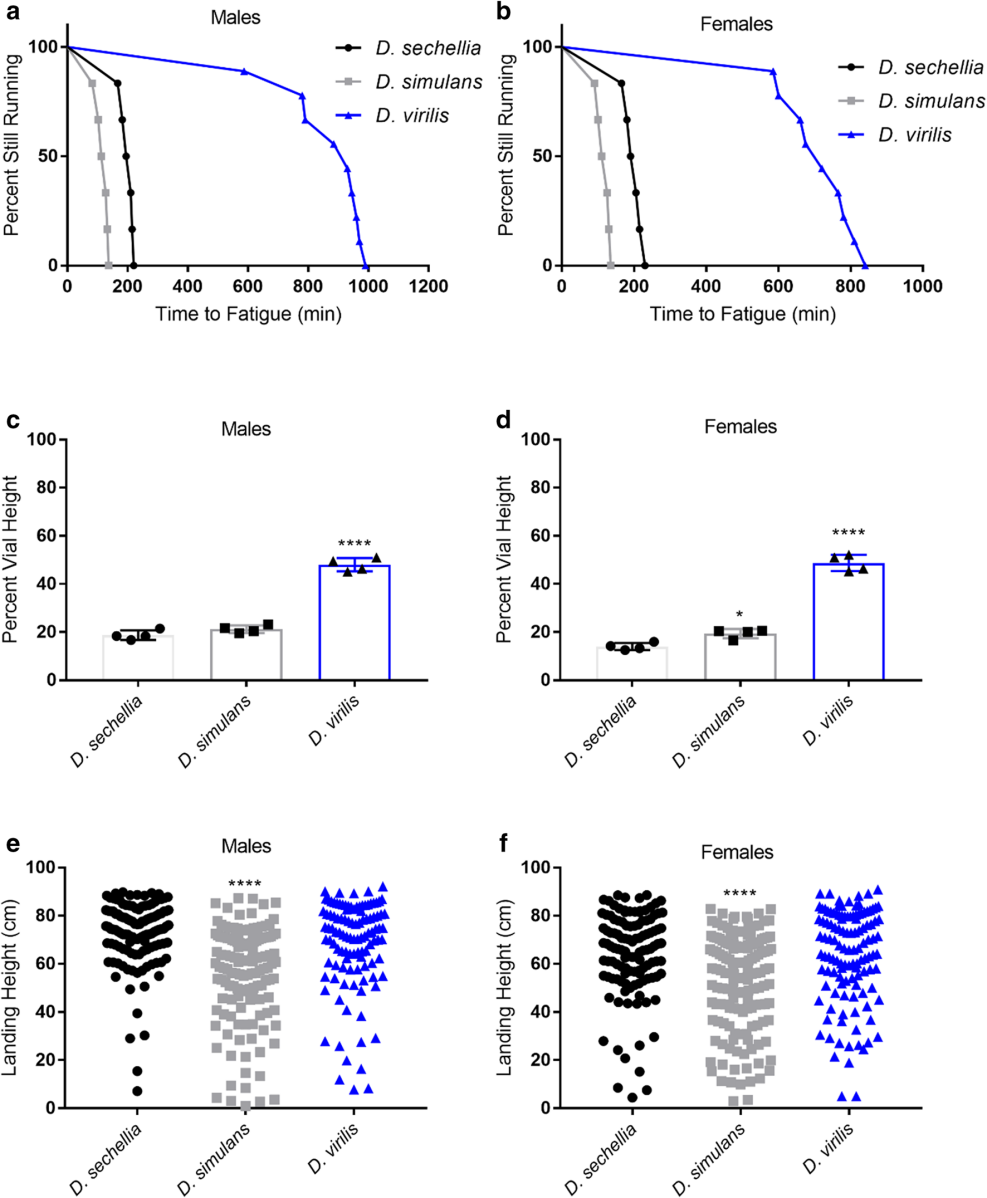
Baseline mobility. **a**, **b** Baseline running endurance. *n* = at least 6 vials for each group. **c**, **d** Baseline climbing speed. *n* = 4 for each group. Each data point in scatter plot represents the average climbing height from a separate replication of 100 flies and is represented as mean ± SD. **e**, **f** Baseline acute flight ability. *n* > 110 each group. All climbing and flight experiments were separately replicated with a different cohort of all groups, with similar results. Asterisks indicate significance. *p < 0.05, ****p < 0.0001

We first assessed differences in baseline endurance, climbing speed, and flight ability. To test endurance, we subjected flies to repeated negative geotaxis until fatigue using a machine called the Power Tower (see “Methods”). *D. virilis* males ran significantly longer than both *D. sechellia* and *D. simulans* males (*p* < 0.0001; log-rank test). *D. sechellia* males ran significantly longer than *D. simulans* (*p* = 0.0005; log-rank test) (Fig. 1a). Females followed a similar rank order (*D. virilis* vs *D. sechellia*, *p* < 0.0001; *D. sechellia* vs *D. simulans*, *p* = 0.0005; log-rank test) (Fig. 1b).

We then measured climbing speed by recording distance climbed in an empty vial in two seconds. Similar to the endurance assay, *D. virilis* males climbed significantly faster than *D. sechellia* and *D. simulans* males (*p* < 0.0001; one-way ANOVA) (Fig. 1c). There was no difference in climbing speed between *D. sechellia* and *D. simulans*. This trend was also observed in females. Female *D. virilis* climbed faster than both *D. sechellia* and *D. simulans* (*p* < 0.0001; one-way ANOVA) but *D. simulans* climbed significantly faster than *D. sechellia* (*p* = 0.02) (Fig. 1d).

Lastly, we tested acute flight ability by dropping flies into a tube containing a polycarbonate sheet coated with Tangle-Trap. Flies that land higher in the tube are considered to have better flight ability than those that land further down. *D. sechellia* and *D. virilis* males had higher landing height compared to *D. simulans* (*p* < 0.0001; one-way ANOVA) (Fig. 1e). Similarly, *D. sechellia* and *D. virilis* females landed higher than *D. simulans* females (*p* < 0.0001) (Fig. 1f).

**Fig. 2:**
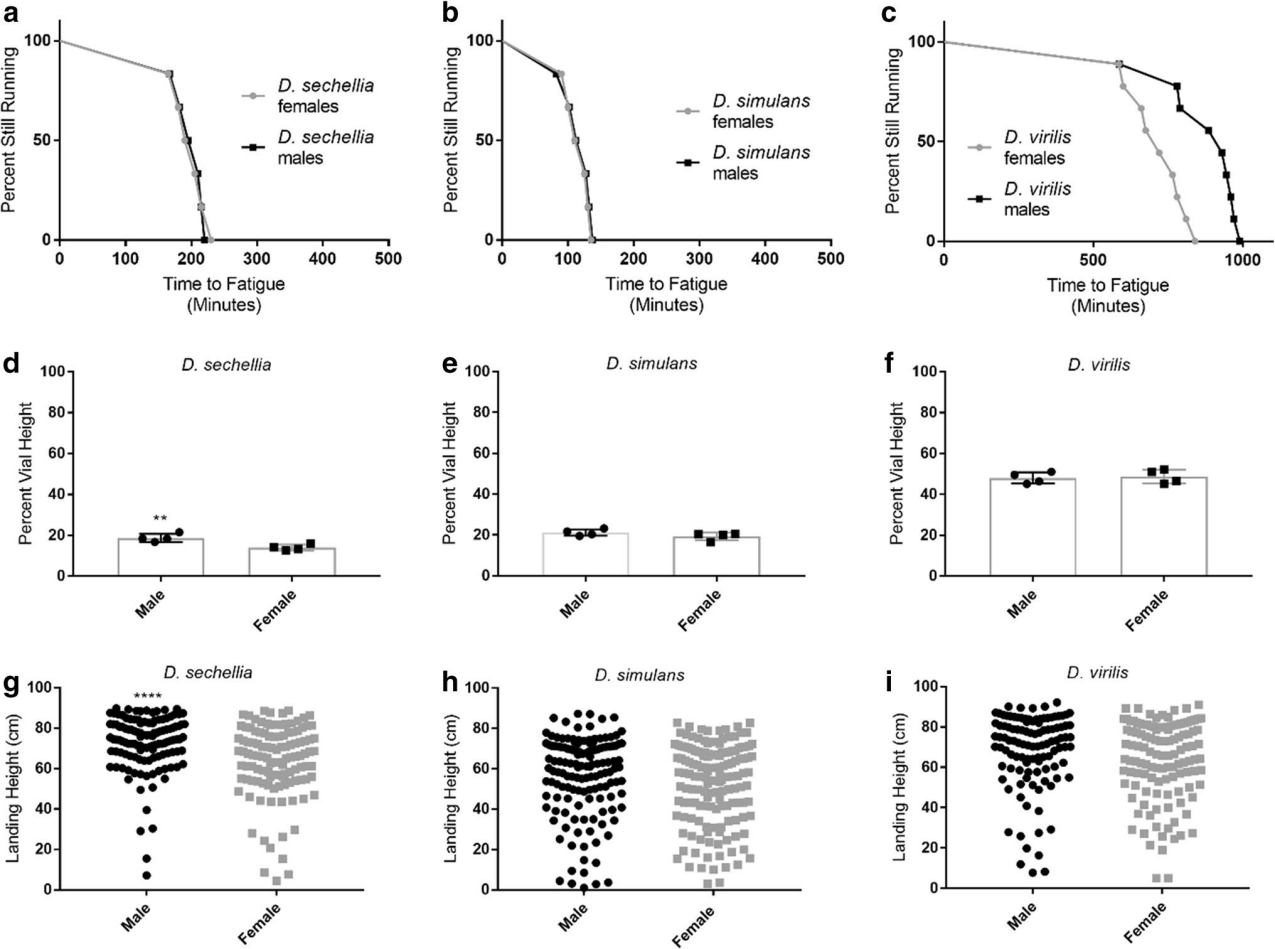
Sex differences in baseline mobility. Data are replotted from same experiments as Fig. 1 to facilitate comparison between sexes. **a–c** Baseline endurance. **d–f** Baseline climbing speed. **g–i** Flight ability. Asterisks indicate significance. **p < 0.01, ****p < 0.0001

We next investigated sex differences within each species. To better visualize this, we replotted the data from Fig. 1. There were no sex differences in endurance in either *D. sechellia* or *D. simulans* (Fig. 2a, b). However, *D. virilis* males ran significantly longer than females (*p* = 0.029; log-rank test) (Fig. 2c). *D. sechellia* males climbed significantly faster than females (*p* = 0.008; Student’s *t* test) (Fig. 2d). There were no sex differences in climbing speed for *D. simulans* or *D. virilis* (Fig. 2e, f). *D. sechellia* males performed better in flight than females (*p* < 0.0001; Student’s *t* test) (Fig. 2g), while there were no sex differences in *D. simulans* (Fig. 2g, h). There were no sex differences in flight in *D. virilis* but the males showed a trend toward better flight that did not reach significance (*p* = 0.067) (Fig. 2i).

#### Response to exercise training

**Fig. 3:**
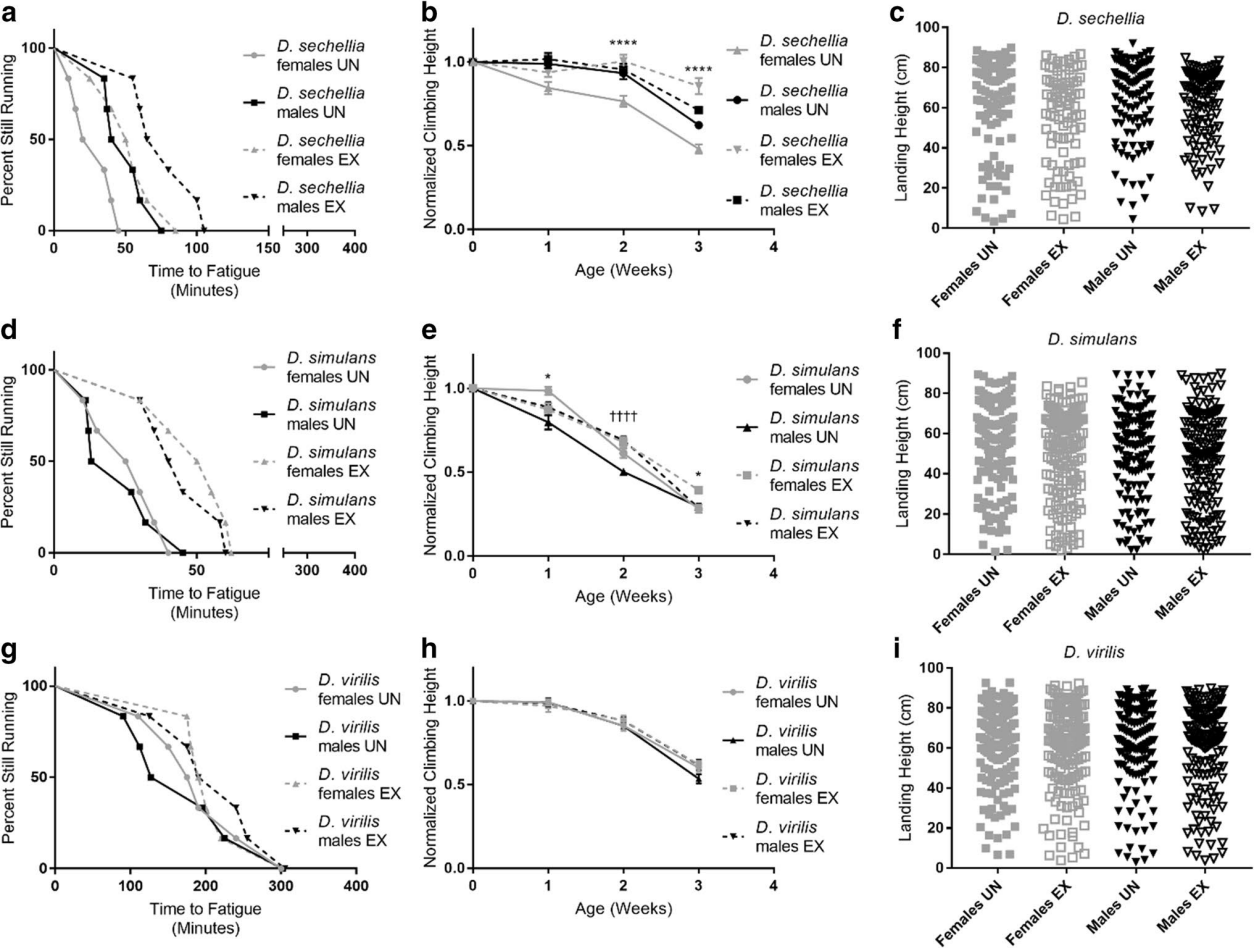
Effects of exercise training on endurance, climbing speed, and flight. **a–c** Male and female *D. sechellia*, **d–f***D. simulans*, and **g–i***D. virilis*. Longitudinal climbing speed is represented as mean ± SEM. Asterisks indicate significance between exercised and unexercised females. *p < 0.05, ****p < 0.0001. Crosses indicate significance between exercised and unexercised males. ^††††^*p* < 0.0001

Since there were discernable interspecific differences in baseline mobility, we next assessed the response to chronic exercise. To test this, each species was subjected to 3 weeks of a ramped exercise training protocol. We assessed climbing speed five times weekly during the 3 weeks. Upon completion of the exercise protocol, we measured endurance and flight performance. Exercised male and female *D. sechellia* ran significantly longer after exercise compared to unexercised controls (male, *p* = 0.02; female, *p* = 0.015; log-rank test) (Fig. 3a). However, only exercised females had faster climbing speed than unexercised controls in weeks two and three (*p* < 0.0001; *n* = 100; two-way ANOVA) (Fig. 3b). Exercise had no effect on flight performance for either males or females (Fig. 3c). Exercised male and female *D. simulans* ran longer than unexercised controls following exercise training (male, *p* = 0.035; female, *p* = 0.007; log-rank test) (Fig. 3d). Exercised females had faster climbing speed in weeks 1 and 3 compared to unexercised (*p* = 0.03; *n* = 100; two-way ANOVA). Exercised males climbed significantly faster than unexercised controls during week 2 but this effect was no longer seen by week 3 (*p* < 0.0001; *n* = 100; two-way ANOVA) (Fig. 3e). Exercise did not affect flight performance. (Fig. 3f). Exercise training had no effect on endurance, climbing speed, or flight performance for *D. virilis* (Fig. 3g–i), perhaps because their baseline mobility is so high.

**Fig. 4:**
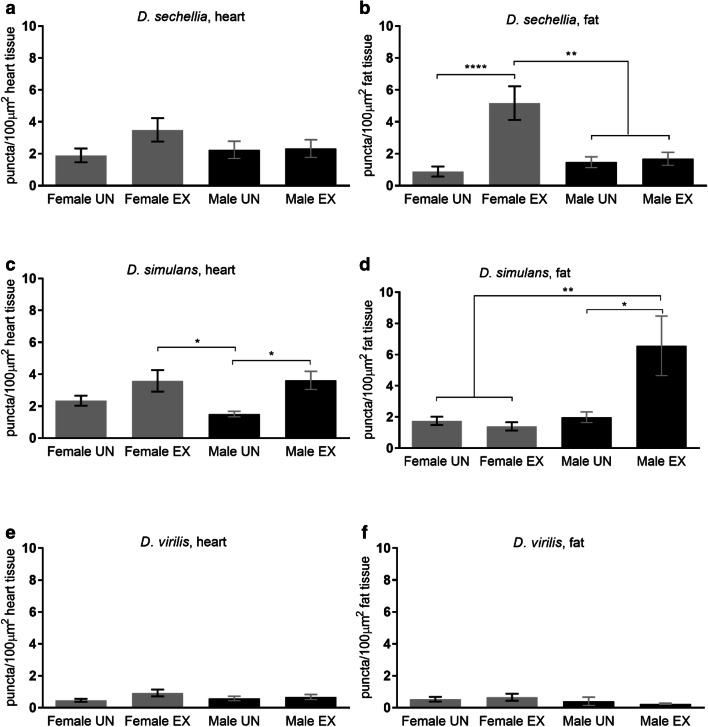
Effects of exercise training on lysosomal activity in heart tissue and fat bodies for **a**, **b***D. sechellia*, **c**, **d***D. simulans*, and **e**, **f***D. virilis*. *n* = 10 for each group. Data are represented as mean ± SEM. Asterisks indicate significance. *p < 0.05, **p < 0.01, ****p < 0.0001

Autophagy and other processes involving lysosomal activity are increased following endurance exercise in mammals and *D. melanogaster* (Sujkowski et al. 2012; Sanchez et al. 2014). To test if this holds true in other *Drosophila* species, we used LysoTracker to measure acidic vesicle formation in the hearts and fat bodies of each species following chronic exercise. LysoTracker staining in these tissues reflects levels of general lysosomal activity, without distinguishing between autophagy, lipolysis or other lysosomal function. Exercise training had no effect on LysoTracker activity in the hearts of *D. sechellia*. However, *D. sechellia* females had higher LysoTracker activity in fat bodies following exercise (*p* < 0.0001; one-way ANOVA) (Fig. 4a, b). *D. simulans* males had greater LysoTracker activity in both heart tissue and fat bodies following exercise training (heart, *p* = 0.019; fat bodies, *p* = 0.014; one-way ANOVA). Exercise did not affect LysoTracker activity in either tissue of females (Fig. 4c, d). Exercise had no effect on LysoTracker activity in hearts or fat bodies of *D. virilis* flies (Fig. 4e, f), consistent with their lack of mobility improvement following exercise.

#### Tyramine and octopamine levels predict baseline speed and endurance in males

**Fig. 5:**
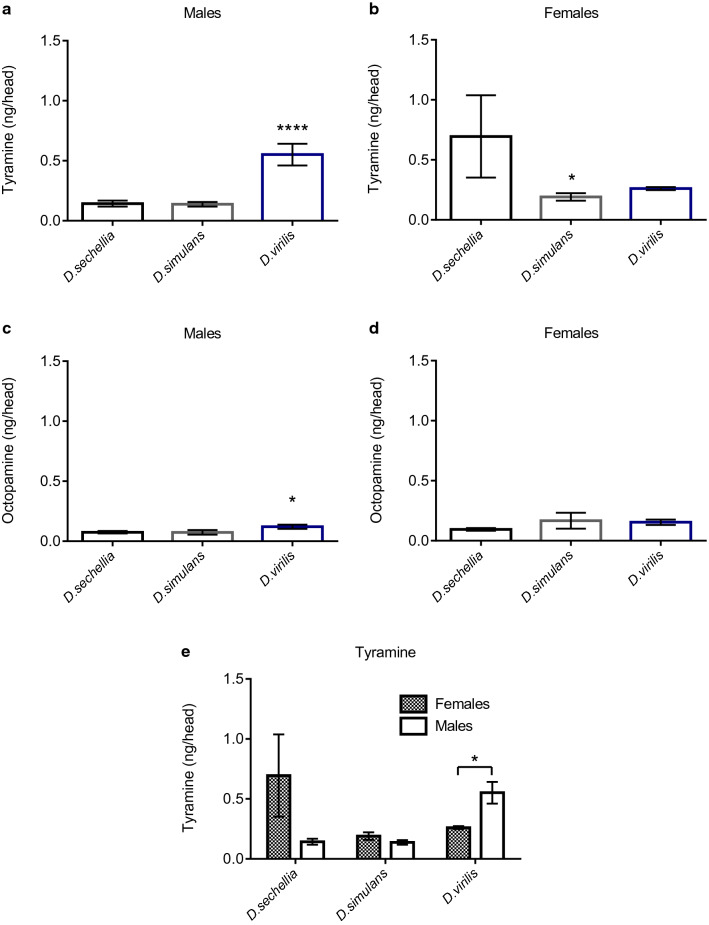
Mass spectrometric measurements of OA and TA. **a**, **b** TA levels in males and females. **c**, **d** OA levels in males and females. **e** Combined male and female TA levels. *n* = 3 for each group and each sample consisted of 5 pooled heads. Data are represented as mean ± SD. Effect sizes, confidence intervals, and raw data are given in the supplementary material. Asterisks indicate significance. *p < 0.05, ****p < 0.0001

Since OA regulates many physiological processes such as metabolism, locomotion, and stress (Hirashima et al. 2000; Hardie et al. 2007; Yang et al. 2015; Li et al. 2017), and is a key mediator of the exercise response (Sujkowski et al. 2017), we asked if differences in baseline mobility and the response to exercise could be attributed to neuronal levels of OA and its precursor, tyramine (TA). We performed quantitative LC–ESI–MS/MS on pooled heads of each species and found that OA and TA levels in males, but not females, predict the differences observed in baseline climbing speed (Fig. 5a, c). *D. virilis* males had higher head OA and TA levels as compared to the males of *D. simulans* and *D. sechellia* (*p* < 0.001 for TA and *p* < 0.05 for OA; one-way ANOVA). Further, the sex-specific differences in baseline endurance had the same rank order as TA levels in the different species (Fig. 5e–g). There were no differences in TA levels between males and females of *D. simulans* and *D. sechellia*, but the males of *D. virilis* had significantly higher TA levels than the females (*p* = 0.029, Welch two-sample *t* test). We found no sex-specific differences in the OA levels of the different species (data not shown). However, neither OA nor TA levels predicted the degree of response to chronic exercise, as *D. virilis,* with high levels of OA and TA, and high baseline speed and endurance, did not improve with exercise (Fig. 3g–i).

## Discussion

Male *D. melanogaster* respond to exercise training with improved endurance, climbing speed, and flight ability (Sujkowski et al. 2017). To determine if this holds true in other *Drosophila* species, we exercise trained *D. sechellia*, *D. simulans*, and *D. virilis* and assessed their response to exercise using mobility assays as outputs. We then asked if any observed differences could be predicted by neuronal baseline OA levels.

Our findings show that there are significant interspecific differences in baseline mobility. *D. virilis* had superior endurance and climbing speed compared to the other species. *D. sechellia* and *D. simulans* performed similarly in endurance and climbing speed, with *D. sechellia* slightly higher in some assays. These results are consistent with a previous study that found similar locomotion patterns between *D. sechellia* and *D. simulans* (Berman et al. 2014). Since both species arose from a common ancestor in the Indian Ocean approximately 300–500 thousand years ago (Lachaise and Silvain 2004), it is not surprising that these two species behave similarly compared to the more distant *D. virilis.* Our previous findings put *D. melanogaster* at the middle of these two extremes. *D. virilis* have higher baseline endurance and climbing speed than most wild-type *D. melanogaster* whereas *D. sechellia* and *D. simulans* have lower. Depending on the wild-type strain, young *D. melanogaster* run approximately 300–1000 min (Sujkowski et al. 2017). However, it should be noted that we only used a single line of the three species in this study and it is possible that other strains may run differently.

Flight performance varied independently from endurance and climbing speed. However, a limitation to this assay is that it tests reflexive flight ability and not long-duration, sustained flight. There is evidence of interspecific gas exchange differences during rest, running, and flight. Running speed is positively correlated with CO_2_ production in *D. virilis*, suggesting a shift in substrate utilization. Similarly, as force production during flight increases, there is a linear increase in the production of CO_2_ in *D. melanogaster* (Lehmann and Schützner 2010). Peak flight performance requires the use of all spiracles for optimal gas exchange and is influenced by the buffer rate of CO_2_ (Heymann and Lehmann 2006). While the mechanisms underlying these interspecific differences in mobility are likely multifactorial, respiration may be one important factor in optimal performance.

The response to exercise training in *D. melanogaster* is sexually dimorphic (Sujkowski et al. 2017). The findings in the current study reveal few sex differences in baseline mobility in other *Drosophila* species. Furthermore, there were no strong sex differences in the response to exercise. Both male and female *D. sechellia* and *D. simulans* flies have higher endurance after exercise whereas *D. virilis* gained no benefit from exercise training.

In *D. melanogaster*, only males benefit from exercise training. However, adult-specific knockdown of *transformer *(*tra*) in the neurons of females permits exercise adaptations (Sujkowski et al. 2017). This means that anatomical differences in the number or arrangement of OA-ergic neurons do not account for the sex difference, and may suggest that masculine OA-ergic neurons are activated more efficiently or at a lower threshold during exercise than feminine neurons. Furthermore, the difference must be *tra* dependent. Interspecific genetic analyses of *tra* have revealed a high degree of divergence of *D. sechellia, D. simulans*, *D. virilis* from *D. melanogaster* (O'Neil and Belote 1992; Kulathinal et al. 2003). It is reasonable to hypothesize that some differences found in the exercise response could be a result of the evolution of the *tra* gene and its expression patterns in the OA-ergic neurons of these species. This phenomenon has been observed in the *fruitless* (*fru*) gene that regulates male-specific sex determination. Expression patterns of *fru* vary between male *Drosophila* species and differences have even been observed in female *D. suzukii* (Yamamoto et al. 2004). Interspecific variance of other known genetic mediators of exercise adaptations likely play an important role as well. For instance, adaptation to thermal stress in *D. simulans* resulted in the modulation of Sestrin and AMPK/*SNF4Ay*, which are involved in metabolic homeostasis (Mallard et al. 2018). The authors also found similar expression patterns of AMPK in *D. simulans* from latitudes of similar temperatures as their experimental conditions. Sestrin is a critical mediator of exercise adaptations, as it activates AMPK and downregulates TORC1, which collectively increases fatty acid oxidation, mitochondrial biogenesis, and autophagy (Richter and Ruderman 2009; Kim et al. 2020; Ho et al. 2016). *Drosophila* found in different latitudes may, therefore, be at a genetic predisposition to respond differently to exercise training. Although these hypotheses are purely speculative, it is clear that the response to exercise in *Drosophila* is a complex phenomenon and likely involves the coordination of multiple systems.

Octopamine, the invertebrate homolog of norepinephrine, is a monoamine that mediates many behavioral and physiological processes in *Drosophila* (Gruntenko et al. 2007; Crocker and Sehgal 2008; Hoyer et al. 2008; Avila et al. 2012; Iliadi et al. 2017). In mammalian mobility and exercise, norepinephrine is important for the mobilization of fatty acids (Barbosa and Migliorini 1982). Circulating norepinephrine increases to stimulate lipolysis and fatty acid mobilization to sustain the energy demand during prolonged exercise (Thibault et al. 1981; Delamarche et al. 1992). Similarly, OA plays a central role in orchestrating flight initiation and substrate modulation during long-duration flight in locusts (Wang et al. 1990; Pflüger and Duch 2011). Despite these conserved effects of OA, our findings suggest that while baseline OA levels have the same rank order as baseline mobility, they do not predict the ability to adapt to exercise, which is in agreement with our previous findings in *D. melanogaster* (Sujkowski et al. 2017). This provides further support to the theory that the ability to adapt to exercise requires carefully modulated activation of OA-ergic neurons while baseline levels of OA may be less important. We also found that TA levels had the same rank order as baseline mobility, as well as the intraspecific sex differences in endurance. Tyraminergic neurons can quickly shift to synthesizing and releasing OA during changes in an animal’s physiological condition (Kononenko et al. 2009), suggesting that TA levels may also contribute to baseline exercise ability.

There are important limitations to consider in this study. To control for performance, we standardized the diet to a 10% yeast 10% sucrose diet. One caveat to this approach is that this diet may not be ideal for optimal performance in each of the species. Dietary composition has substantial effects on speed and endurance in multiple genotypes of *D. melanogaster* (Bazzell et al. 2013; Lowman et al. 2018) so it is possible that this phenomenon could occur in different *Drosophila* species. We also only tested one genotype of each species. It is possible that different genotypes would respond differently to exercise training as well. Additionally, the low sample size for mass spectrometry measurements of OA and TA limit the ability to draw definitive conclusions of their role in mobility and exercise.

In conclusion, our findings suggest that there are interspecific differences in mobility and response to exercise training. Baseline OA and TA levels had the same rank order as baseline speed and endurance in males across species, but did not predict response to chronic exercise. There were few sex differences in baseline mobility and no sex differences in the response to exercise training. The observed differences in the response to exercise cannot be attributed to baseline OA levels, but could be due to the ability to activate OA-ergic neurons or to differences in other mediators of exercise.

## Electronic supplementary material

Below is the link to the electronic supplementary material.

Supplementary file1 (PDF 127 kb)
