# Supplementary material for: Variation in mobility and exercise adaptations between Drosophila species
Source: J Comp Physiol A Neuroethol Sens Neural Behav Physiol. 2020 Apr 25;206(4):611–21. doi: 10.1007/s00359-020-01421-x (PMC7314734; doi:10.1007/s00359-020-01421-x)
Supplement: Supplementary file 1 — Supplementary file1 (PDF 127 kb) [file 359_2020_1421_MOESM1_ESM.pdf]

Welch Two Sample t-test

| Species | Mean Female | Mean Male | t-statistic | p.value | df | conf.low | conf.high | method | alternative |
|---|---|---|---|---|---|---|---|---|---|
| D.sechellia | 0.695 | 0.143 | 2.779 | 0.107 | 2.022 | -0.294 | 1.398 | Welch Two Sample t-test | two.sided |
| D.simulans | 0.191 | 0.138 | 2.516 | 0.078 | 3.354 | -0.010 | 0.117 | Welch Two Sample t-test | two.sided |
| D.virilis | 0.260 | 0.551 | -5.527 | 0.029 | 2.075 | -0.510 | -0.072 | Welch Two Sample t-test | two.sided |

One-way ANOVA

| Factor | Transmitter | Sex | power | df | sumsq | meansq | statistic | p.value | etasq | partial.etasq | omegasq | partial.omegasq | cohens.f |
|---|---|---|---|---|---|---|---|---|---|---|---|---|---|
| Species | Tyramine | Male | 1 | 2 | 0.338 | 0.169 | 55.168 | 0 | 0.948 | 0.948 | 0.923 | 0.923 | 4.288 |
| Species | Tyramine | Female | 0.809 | 2 | 0.448 | 0.224 | 5.653 | 0.042 | 0.653 | 0.653 | 0.508 | 0.508 | 1.373 |
| Species | Octopamine | Male | 0.93 | 2 | 0.004 | 0.002 | 8.249 | 0.019 | 0.733 | 0.733 | 0.617 | 0.617 | 1.658 |
| Species | Octopamine | Female | 0.492 | 2 | 0.009 | 0.005 | 2.71 | 0.145 | 0.475 | 0.475 | 0.275 | 0.275 | 0.95 |

Raw data

| **Tyramine** | | ***D.sechellia*** | | | ***D.simulans*** | | | ***D.virilis*** | |
|---|---|---|---|---|---|---|---|---|---|
| Female | 0.629276 | 0.3893 | 1.066063 | 0.167 | 0.1795 | 0.22611 | 0.246647 | 0.26249 | 0.271127 |
| Male | 0.126425 | 0.1302 | 0.172032 | 0.1282 | 0.1599 | 0.1245 | 0.62894 | 0.45205 | 0.57277 |
| | | | | | | | | | |
| **Octopamine** | | ***D.sechellia*** | | | ***D.simulans*** | | | ***D.virilis*** | |
| Female | 0.082129 | 0.1026 | 0.098491 | 0.0936 | 0.2215 | 0.18661 | 0.134009 | 0.1791 | 0.149653 |
| Male | 0.062575 | 0.0784 | 0.083885 | 0.0607 | 0.0643 | 0.09546 | 0.115652 | 0.14008 | 0.106553 |
